# Evaluating a South African mobile application for healthcare professionals to improve diagnosis and notification of pesticide poisonings

**DOI:** 10.1186/s12911-019-0791-2

**Published:** 2019-03-11

**Authors:** Siti Kabanda, Hanna-Andrea Rother

**Affiliations:** Environmental Health Division & Centre for Environmental and Occupational Health Research (CEOHR), School of Public Health and Family Medicine, University of Cape Town; Faculty of Health Sciences, Falmouth Building, Anzio Road, Observatory, Cape Town, 7925 South Africa

**Keywords:** Acute pesticide poisoning, Healthcare professional, Low-and middle-income countries, mHealth, Pesticide notification, South Africa, Pesticides, Health promotion

## Abstract

**Background:**

Mobile health is a fast-developing field. The use of mobile health applications by healthcare professionals (HCPs) globally has increased considerably. While several studies in high income countries have investigated the use of mobile applications by HCPs in clinical practice, few have been conducted in low- and middle-income countries. The University of Cape Town developed a pesticide notification guideline which has been adapted and embedded into a South African Essential Medical Guidance mobile application. This study evaluated the usefulness of the guideline within a mobile application for improving the ability of HCPs to diagnose and notify on acute pesticide poisonings (APPs).

**Methods:**

A descriptive online questionnaire, with 15 open- and 20 closed-ended questions, was completed by 50 South African emergency medicine physicians and registrars (i.e. medical doctors training as specialists) between December 2015 to February 2016. Descriptive statistics were used to calculate response frequencies and percentages using SPSS version 23. Texts from the open-ended questions were thematically analysed. Fisher’s exact test was applied to determine associations.

**Results:**

A significant association was found between participants’ knowledge that APP is a notifiable condition, and ever reporting the poisoning to the National Department of Health (*p* = 0.005). Thirty four percent of the participants were aware of the guideline within the Essential Medical Guidance application despite only seven participants having used it. Those who used the guideline found it provided useful information for the identification of unlabelled pesticides products and promoted reporting these cases to the National Department of Health for surveillance purposes. In addition, it appeared to facilitate the prompt diagnosis and treatment of APP cases, and most intended to continue using it for training and educational purposes.

**Conclusions:**

Mobile health applications appear to support overburdened medical education programmes and promote better patient care. However, since most participants were not aware of the existence of the pesticide guideline within the studied essential medicine application, there is potential for the use of healthcare applications to play a more central role in healthcare systems and medical training. Furthermore, the field of medical informatics could support HCPs through mobile applications in improving reporting of APP.

## Background

Worldwide approximately three million acute pesticide poisoning (APP) cases occur yearly [1] and yet this is a neglected public health concern. In particular, APPs are a widespread problem in World Bank classified low- and middle-income countries (LMICs), including South Africa (SA) as an upper middle-income country. In the sub-Saharan Africa context, SA is the biggest user of pesticides in both occupational and non-occupational settings, where pesticide use is extensive and pesticide exposures are common [2–4]. In SA, an APP is a notifiable medical condition under the National Health Act [5], and by law healthcare professionals (HCPs) are required to notify poisonings from any pesticide to the National Department of Health (NDOH) [2, 4]. Despite this, cases of poisoning continue to be under-reported. The assumption is that health professionals have received adequate training in medical school to diagnose and treat pesticide poisonings from all types of pesticides. The reality is that medical curricula are extremely dense and pesticide poisoning is not a priority area. What is needed are innovative methods for providing APP information to HCPs. To address this challenge, this study aimed to assess the use of mobile health (mHealth) application for APPs (i.e. the diagnosis and notification of APPs) to provide supplementary information to HCPs.

Notification of an APP is an important surveillance tool for controlling and decision making to reduce the harmful effects of pesticides, particularly from street pesticides (i.e., agricultural pesticides and other pesticides sold illegally mostly in communities with low socio-economic status for domestic pest control) [4]. The importance of this notification system is to alert local and national health services of the pesticides that are causing morbidity or mortality, and which require to take appropriate prevention responses. Furthermore, as a signatory to the Rotterdam Convention, South Africa is required to submit quarterly reports of pesticide poisonings. Many cases, however, go unreported leading to poor surveillance data. In SA, it is estimated that only 10 to 20% of hospitalized cases are reported [2]. A similar magnitude of under-reporting has also been observed in Tanzania where a study found that 78% of APP cases presented to health facilities go unreported [6]. This is often to limited information on pesticide poisoning leading to incorrect diagnosis and treatment, as in turn, under-reporting [4]. For example, many APP symptoms present as flu-like symptoms [7]. Furthermore, there is knowledge gap regarding classes of pesticides and reporting requirements. For example, only organophosphate-containing pesticides (OPs) tend to be reported, while other pesticide classes are not, such as pyrethroids, carbamates, organochlorines and rodenticides [8, 9].

Studies assessing the adequacy of HCPs training in both high-income countries and LMICs on environmental health risks have found that most HCPs have limited knowledge in diagnosing and managing environmental exposure problems, such as lead poisoning and APP [10–14]. This is primarily because many medical schools offer little or no training in environmental health, as it is not considered a priority area in what are usually already full medical curricula [15, 16]. As a result, many HCPs do not conduct an environmental exposure history, which is key in making a diagnosis of APP [14]. Environmental history taking should be included when general history taking is conducted as part of a standard diagnostic process when a patient is first seen by an HCP. For example, the HCP could use the CH^2^OPD^2^ mnemonic–that is; asking questions about community, home, hobbies, occupation, personal habits, diet and drugs [17] – to find out if, for example, the patient lives in a community with high use of street pesticides.

Another challenge, often faced by HCPs in South Africa, is the difficulty of linking APP to illegal or unlabelled street pesticides [18]. Many poor urban residents rely on cheap, easily accessible, and often highly toxic agricultural pesticides that have been decanted into unlabelled containers for domestic pest control [4]. When an exposed individual is taken for medical care, it becomes difficult for HCPs to diagnose poisonings from exposure to unlabelled products and complicates their ability to comply with notification requirements. Another challenge is that there is a scarcity of easily accessible resources to assist busy HCPs with APP diagnosis, treatment and notification [4].

mHealth applications present a unique platform to supplement medical education on environmental health topics generally, and to assist HCPs in identifying and notifying APP cases in particular. mHealth, the utilisation of mobile technology in health care, has the potential to support the provision of high-quality health care services and keep HCPs up to date, particularly in LMICs [19, 20]. This is due to high coverage of mobile devices. For example, in SA almost all HCPs have cell phones and therefore implementation of mobile application could possibly alleviate the overburdened health care delivery systems in these countries [21]. Therefore, this study provides an insight of how mHealth could support SA, where there is excessive use of pesticides and exposure with relatively well-developed health system and high cell phone ownership levels - but continuing underreporting of APPs.

In recent years, the use of mobile phones and other mobile devices by HCPs in their clinical practice has been increasing. This has led to the rapid development of medical-related mobile applications, including those for the management and monitoring of patients with different illnesses, clinical decision-making resources, and continuing medical education on specific topics, among others [22–28]. Despite this development, there are limited studies that have evaluated mHealth tools targeted at HCPs [29, 30].

A key aspect in evaluating the usefulness of mHealth is understanding an HCP’s attitude towards the use of mobile applications. Several studies have examined the use of mobile applications by HCPs and have shown that generally they perceive mobile applications to be essential and effective tools to support their day-to-day work [31–33]. For example, a study conducted in the United Kingdom (UK) evaluated a hospital-specific smartphone application (i.e., iTreat) used for antibiotic selection and clinical management of infections. The study found that most doctors felt the application was an effective tool because it reduced the need to refer to cumbersome clinical manuals, thus saving time during clinical rounds [32]. In low- and middle-income countries, such studies are needed to determine effective mHealth strategies (i.e. use of behavioural theories [21]) for large-scale use. Thus, for production of mHealth applications, developers will be able to access evidence-based information on the usefulness, as well as the disadvantages and/or advantages of the applications to improve on functionality [34]. To enhance South African HCPs’ capacity to diagnose and report APPs, the University of Cape Town (UCT)’s Division of Environmental Health, and Centre for Environmental and Occupational Health Research (CEOHR), together with other stakeholders (academic researchers, NGOs and the SA government), developed a Pesticide Notification Guideline (PNG) [4]. The guideline is composed of an algorithm that aims to help HCPs improve notification of APPs, particularly from street pesticides. Included in this guideline is a point chart that contains pictures of common unlabelled street pesticides for family caregivers or patients to indicate the pesticide product responsible for the poisoning.

Using behavioural theories (Theory of Planned Behaviour and Social Cognitive Theory), this study evaluated the PNG within the EM Guidance mobile application to improve the ability of HCPs to diagnose and reporting of APP cases. Furthermore, assessing the improvement of their understanding that APP is a notifiable medical condition. In addition, the study assessed the ways in which the PNG could be improved to enhance its effectiveness and applicability.

## Methods

### Study design and sampling

A descriptive survey was conducted with 50 South African emergency medicine registrars (i.e., general medical doctors training as specialists such as EM specialists/physicians) and physicians from December 2015 to February 2016. An online consent form and questionnaire with closed- and open-ended questions was used to assess the use and impact of the PNG within the EM Guidance mobile application. Participation in this study was anonymous and voluntary. Participants were identified through the SA Medpages Directory website that lists emergency medicine registrars and physicians practicing throughout SA in the nine provinces. Other participants were identified by an emergency medicine physician key informant based in Cape Town, in the Western Cape Province. This physician circulated the survey details to emergency medicine physicians and registrars working in the Western Cape Province on behalf of the study researchers and posted the study request on the SA emergency medicine website. Willing participants were emailed the survey link by the first author. Incomplete questionnaires and participants who were not emergency medicine registrars and physicians were excluded from the study. A purposive sampling method was used to select emergency medicine registrars and physicians working in SA of varying seniority, and who were willing to participate in the study. This occupational eligibility requirement was chosen because the EM Guidance mobile application was originally designed for these professionals who are often the first to see a patient with APP. Recruitment strategies included sending emails and making phone calls to seventy-three potential participants.

Ethics approval was granted from the Faculty of Health Sciences Human Research Ethics Committee (HREC REF: 753/2015) at the University of Cape Town, South Africa prior to data collection.

### Guideline design

**Fig. 1:**
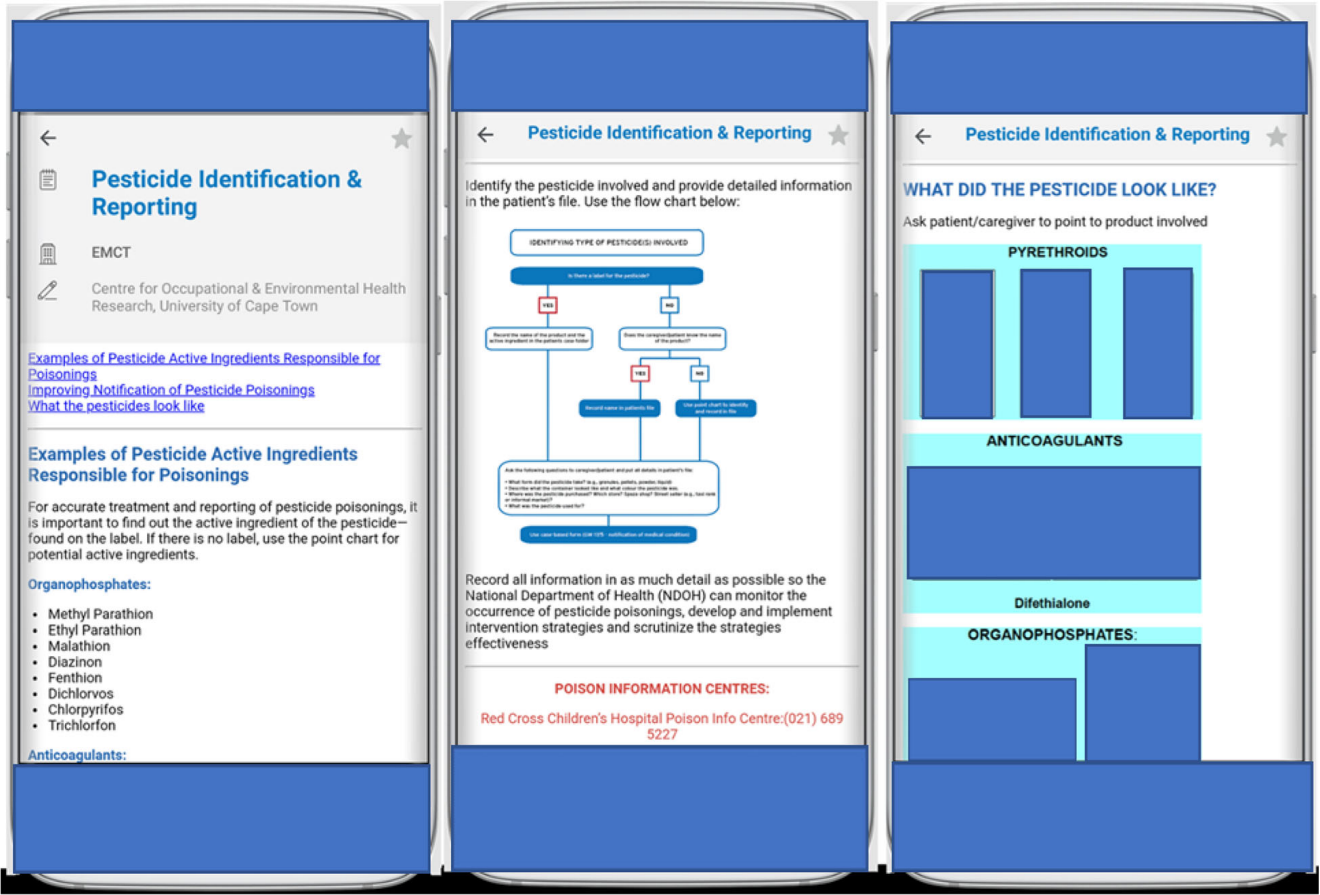
Screen shot of UCT’s pesticide notification guideline within the EM Guidance mobile application. Reproduced with the permission of Essential Medical Guidance team website [36]. The pesticide photos were taken by HA Rother and the CEOHR team. The photos show various examples of pesticides (i.e. pyrethroids, anticoagulants and organophosphates) and those decanted in unlabelled bottles

The PNG was originally produced as printed material and 20,000 copies were distributed to HCPs in the Western Cape Province of South Africa. In 2014, this guideline) was adapted into a South African Essential Medical (EM) Guidance mobile application entitled “EM Guidance” (see Fig. 1) [35]. The EM Guidance mHealth application was developed by the EM Guidance Medicines team and their contributors. It is freely available for download from the EM Guidance website [36] and advertised on the South African EM Facebook page. The application was downloaded 9243 and 146,832 times in South Africa and worldwide respectively, between February 2014 and January 2016. Furthermore, with recent changes to the application, there were 28,615 downloads in SA and 16,810 outside SA from January 2017 to October 2018.

To evaluate the PNG as part of a mHealth application (which will be termed “PNG-application” for the sake of this paper), the Theory of Planned Behaviour (TPB) [37] and Social Cognitive Theory (SCT) [38] were used to assess healthcare professionals’ behaviour [39–41]. According to the TPB, the most important determinant of an individual’s behaviour is intention. The intention, furthermore, is influenced by three constructs: attitude, subjective norm (a person’s opinion about another’s anticipated behaviour) and perceived behavioural control (perceived ease of performing the behaviour) [37]. The SCT provides guidance on understanding different factors (i.e. personal, environmental and human behaviour) that influence HCPs in adopting a behaviour (in this case the use of PNG within the EM Guidance mobile application). According to SCT, self-efficacy is regarded as the most important motivation for behavioural change [38] and was one of the constructs assessed in this study.

### Survey instrument

The questionnaire was developed based on concepts from the Theory of Planned Behaviour (TPB), Social Cognitive Theory (SCT) and relevant literature identified to assess factors influencing the use of PNG-application [32]. This questionnaire was created using the web-based survey software, SurveyMonkey®, to accommodate HCPs’ busy clinical schedules and multiple locations. An online survey method was used because it is faster to complete and has been shown to have a better return rate with HCPs than paper-based surveys [42].

Before it was administered, the questionnaire was piloted with five physicians. This was to ensure the validity and reliability of the questionnaire. The questionnaire was then updated based on these responses to address misunderstandings, repetition or incomplete information provided. The final survey questionnaire consisted of 20 closed- and 15 open-ended questions addressing demographic characteristics, and knowledge of and clinical practice and experience with APP. It captured information on general use of medical mobile applications, including perceived advantages and disadvantages of using the PNG-application, and asked for recommendations for improving it.

### Data analysis

Data was captured on Microsoft Excel for cleaning and subsequently into the Statistical Package for Social Sciences (SPSS) version 23, for analysis. Descriptive statistics were used to calculate response frequencies and percentages. Due to the small sample size and low expected values, a Fisher’s exact test was used to determine any specific associations between variables. For meaningful interpretation of the survey responses to the 5-point Likert scale, these were collapsed into three categories: agree, uncertain (this implies neither disagree nor agree) and disagree. For all statistical tests, a *p*-value of < 0.05 was considered statistically significant. Since participants provided multiple hospital names for work location, to simplify the analysis the data were categorized into hospital type (i.e. public and private hospitals). There was no implication of hospital type in the final analysis since this was not a question asked and answers were provided by some participants. In South Africa, the bulk of APP cases would generally be referred to or presented at public hospitals for poisonings from poor communities or workers.

Responses to open-ended questions were manually analysed thematically, which involved extensive familiarisation with the data by reading and re-reading the responses and identifying key ideas [43]. In addition, themes or patterns based on relevant literature and concepts from the TPB and SCT were identified and recurrent topics raised in the data were explored. Certain responses to some of the open-ended questions were coded as quantitative data in Microsoft Excel for calculating frequency counts or percentages [44]. Quotations are included in this journal article to illustrate the common themes and experiences of the participants.

## Results

### Demographic information

A total of 58 of the 73 emergency medicine physicians and registrars contacted clicked on the anonymous online survey link. Of these, 50 successfully completed the surveys; six were incomplete, one respondent declined to participate, and one did not fulfil the inclusion criteria. The response rate was, therefore, 69% (50 of 73). Although this was a general survey across the country, most of the participants were from five of the nine provinces in SA – that is; the Western Cape, Limpopo, Gauteng, Kwazulu-Natal and the Northern Cape.

The demographic profile of participants is listed in Table 1. Sixty-eight percent of participants were between 30 and 39 years of age with a male predominance (68%); 60% were emergency physicians and 40% were registrars. Nearly half of the participants had been practicing emergency medicine for one to three years, 82% worked in public hospitals and 64% were based in the Western Cape Province.

**Table 1:** Demographic characteristics of survey participants (*N* = 50)

	n (%)
Age	
18–29	1 (2)
30–39	34 (68)
40–49	14 (28)
50–59	1 (2)
Gender	
Female	16 (32)
Male	34 (68)
Current position	
Emergency medicine registrar	20 (40)
Emergency physician	30 (60)
Years practicing in current position	
Less than year	7 (14)
1–3 years	23 (46)
4–6 years	12 (24)
More than 6 years	8 (16)
Type of hospital currently stationed at	
Public	41 (82)
Private	4 (8)
Other (i.e. at sea on cruise ships and non-emergency medical services)	5 (10)
Province in which the hospital is located	
Gauteng	10 (20)
Western Cape	32 (64)
Kwazulu-Natal	5 (10)
Limpopo	1 (2)
Northern Cape	2 (4)

### Knowledge on pesticide poisonings

**Fig. 2:**
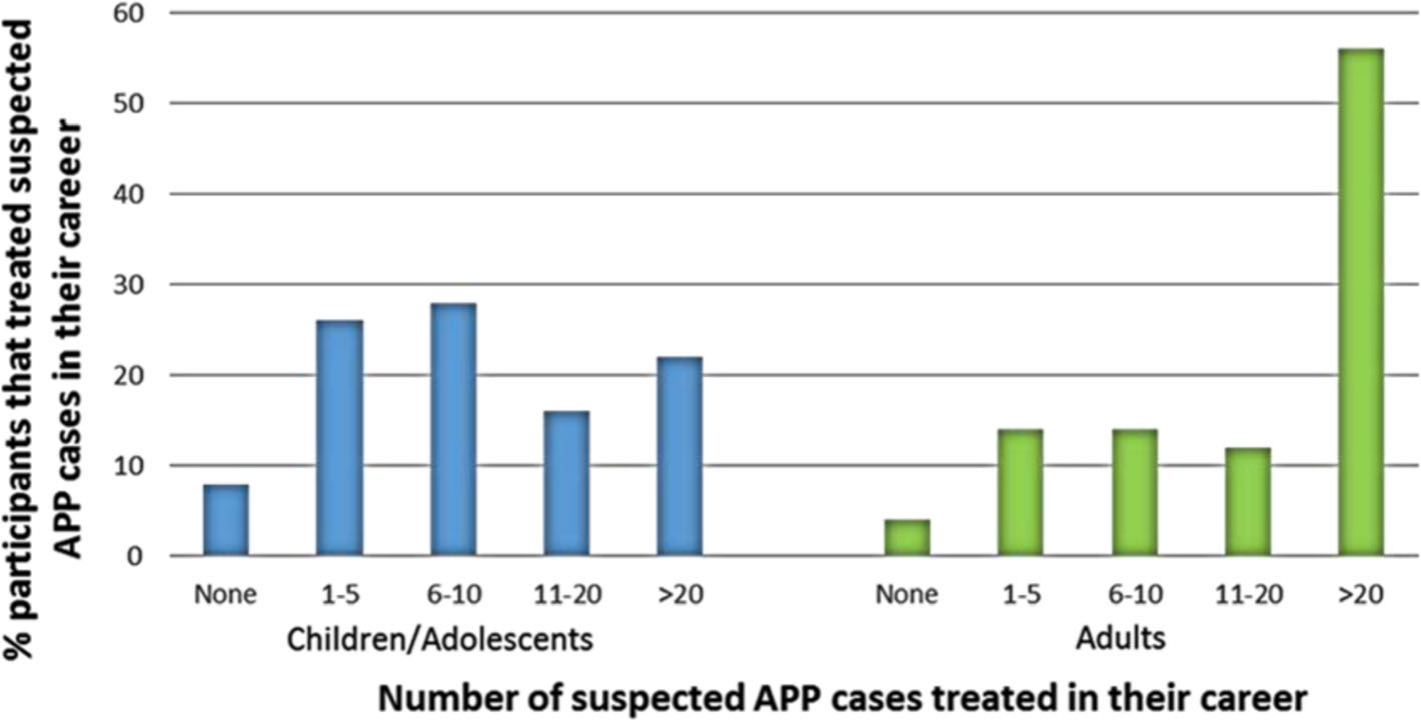
Number of suspected pesticide poisoning cases treated in participants’ career

To understand participants’ history of handling pesticide poisoning cases, they were asked to indicate the number of suspected APP cases they treated and reported in their career. Most participants indicated having encountered patients who may have been exposed to pesticides. This is illustrated by the majority having had treated children or adolescents (92%), and adults (96%) with APPs (Fig. 2). Just over half of the participants (56%) had treated more than twenty adult APP cases in their career.

**Fig. 3:**
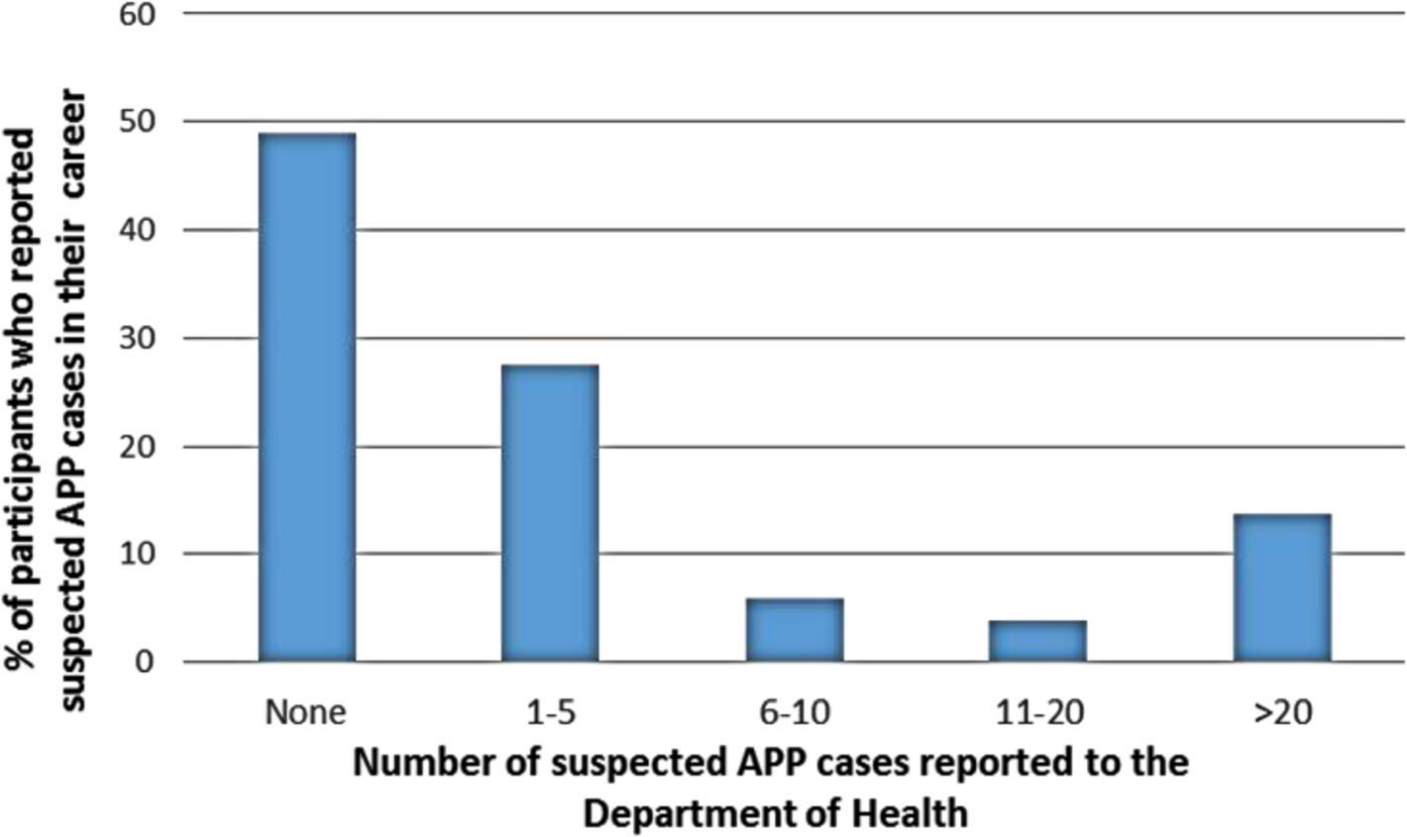
Number of suspected pesticide poisoning cases reported to NDOH in their career

With regard to notification, 72% of participants indicated knowing that APP was a notifiable condition (Table 2) and 68% correctly listed the pesticide classes required to be reported (i.e. carbamates, pyrethroids, organophosphates and anticoagulants). However, results show that nearly half of the participants (48%) had never reported any APP cases to the NDOH despite knowing that APP is a notifiable medical condition (Fig. 3). A significant association was found between participants’ knowledge that APP is a notifiable condition and ever reporting the poisoning to NDOH (*p* = 0.005).

**Table 2:** Participants’ knowledge of pesticide poisonings (*N* = 50)

	Agree n (%)	Uncertain n (%)	Disagree n (%)
Pesticide poisoning is a medical notifiable condition	36 (72)	11 (22)	3 (6)
Poisoning caused by mosquito repellents should be notified to NDOH	24 (48)	18 (36)	8 (16)
After diagnosing and treating poisoned patients, I (the doctor) indicate in the case file that the poisoning should be reported to NDOH	17 (34)	16 (32)	17 (34)

Approximately 28% of the participants reported notifying one to five cases of APP, while about 14% notified more than 20 cases during their career. Differences were identified with regard to reporting of APP caused by certain pesticide product type. For instance, 36% of participants felt uncertain as to whether poisoning caused by mosquito repellents should be notified to the NDOH, 48% indicated reporting for these products was required and 16% disagreed with the statement. Despite 72% indicating that pesticide poisoning is a notifiable medical condition in SA, only 34% were aware of the reporting procedures of APPs to the NDOH, while a further 32% were uncertain.

No significant association was found between pesticide poisoning knowledge and the age or gender of participants. However, there was a significant association between years in the position and knowledge of pesticide poisoning in relation to reporting of pesticide poisoning cases to the NDOH (*p* = 0.018). The results showed that 82% of participants who were in the position of emergency medicine registrar or physician for least four to six years were likely to know that pesticide poisonings need to be reported as compared to those with fewer than four years’ experience (45%) or with more than six years’ experience (25%).

### Participants’ mobile application use

Nearly all the participants reported using mobile devices at work, with most using a smart device particularly an iPhone (65%) (Table 3). Some of the participants used a combination of smart devices and tablets.

**Table 3:** Participants’ use of mobile devices (*N* = 50)

	n (%)
Type of mobile device used while at work	
Use tablet-Android mobile device while at work	5 (7.4)
Use tablet-iPad mobile device while at work	10 (14.7)
Use smartphone-Android mobile device while at work	19 (27.9)
Use Smartphone-iPhone mobile device while at work	32 (47.1)
Do not use any of the above mobile devices while at work	1 (1.5)
Use other mobile devices while at work	1 (1.5)
Number of participants that have medical related applications in their mobile devices	
Yes	46 (92.0)
No	4 (8)
The frequency use among those that own medical related applications	
Daily	31 (67.4)
Weekly	12 (26.1)
Monthly	0 (0)
Occasionally	2 (4.3)
Rarely	1 (2.2)

Most of the participants (92%) had medical related applications installed on their devices (Table 3). Of those that had medical related applications, the majority (67%) used their applications daily, while 26% used them weekly (Table 3). The remaining participants (6.5%) reported using their medical applications either occasionally or rarely.

Among those that used medical-related applications, they agreed that these provided quick access to clinical guidelines (87%) and useful information at point-of-care (89%) (Table 4). This illustrates the popularity of mobile applications in general among HCPs for clinical practice, with most intending to continue using applications for training or educational purposes (87%). The EM Guidance was among the most commonly used medical application (40%), with the majority (80%) aware of the South African EM Guidance application.

**Table 4:** Participants’ perception regarding use of medical related applications (*N* = 46)

	Agree n (%)	Uncertain n (%)	Disagree n (%)
They provide me with quick access to clinical guidelines	40 (86.9)	2 (4.3)	4 (8.7)
They provide useful information at point-of-care	41 (89.1)	1 (2.2)	4 (8.7)
I will continue to use for training/educational purposes	40 (86.9)	1 (2.2)	5 (10.9)

### Awareness and use of PNG-application

Thirteen participants (33%) indicated awareness of the PNG-application, but only seven confirmed having used it. Five of these participants treated more than 20 APP cases with two that treated between 11 to 20 cases. The other six participants who were aware of the PNG-application did not use it because they had not seen any APP cases, or they had only recently downloaded. One participant commented that PNG-application was not user-friendly, without providing further explanation. There was no significant association found between treating APP cases and using the PNG-application. This could possibly be due to the relatively small sample size. The seven participants who used the PNG-application found it suitably designed for use in a medical setting, easily accessible, helpful and user-friendly in the presence of the patient and, indicated a desire to continue using it in the future.

Although awareness of the PNG was low among most participants, understanding the perspectives of the seven who used the PNG-application for APP notification is important because this information will help in improving the PNG-application in the future. It also highlights the need for further training and research. Table 5 shows that those who used the guideline agreed that it aided their ability to report APP cases as required, including for poisonings from street or unlabelled pesticides. This indicated that they had good attitudes towards the guideline. Comments illustrating this included:

**Table 5:** Participants’ attitudes on the pesticide notification guideline for notifying APP (*N* = 7)

	Agree n (%)	Uncertain n (%)	Disagree n (%)
It has aided me with the process for notifying pesticide poisoning cases	4 (57.2)	2 (28.6)	1 (14.3)
It has assisted in improving on the number of pesticide poisoning cased I have reported	3 (42.9)	2 (28.6)	2 (28.6)
It has improved my confidence to report pesticide poisoning cases	4 (57.2)	0.0	3 (42.9)
It has improved my ability to report poisonings from street or unlabelled pesticides	4 (57.2)	1 (14.3)	2 (28.6)

*“I confirmed a pesticide as being a Paraquat - and as a result this altered patient disposition dramatically and resulted in a case being reported to NDOH and farm owner investigated … the guideline has also improved my knowledge on how to report and I also found point chart to be very useful.” [R1]*

*“It has alerted one to the concept of notification and advised on the appropriate form to be completed.” [R3]*

*“Possible improvement in my skills due to easier identification of packaging.” [R6]*

Although the PNG within the EM Guidance mobile application is meant to promote better notification of APPs, it has the potential to also promote more accurate diagnoses. However, only three of the seven participants used the guideline to diagnose APPs.

Five of the seven participants perceived the pesticide notification guideline to have assisted them in identifying unlabelled pesticide products such as carbamates using the point chart. One participant commented:

*“The point chart was shown to patient’s family to help guide us to determine which pesticide patient had ingested.” [R1]*

In another example, one participant mentioned that in some instances, patients bring containers to the hospital. The use of the guideline provided an easy reference to identify unlabelled pesticide products.

Emergency medicine physicians and registrars also mentioned barriers or disadvantages related to the use of the pesticide notification guideline. Some participants felt that the pesticide notification guideline lacked contact information.

*“Not enough information on application. I contact poison information centre if known active ingredient/ name of pesticide.” [R7]*

For others, the concern was with the internet network when downloading the images on the application:

*“If there is poor network, it slows to load images.” [R5]*

*“Potential disadvantage is poor 3G signal.” [R6]*

### Features of the pesticide notification guideline

**Fig. 4:**
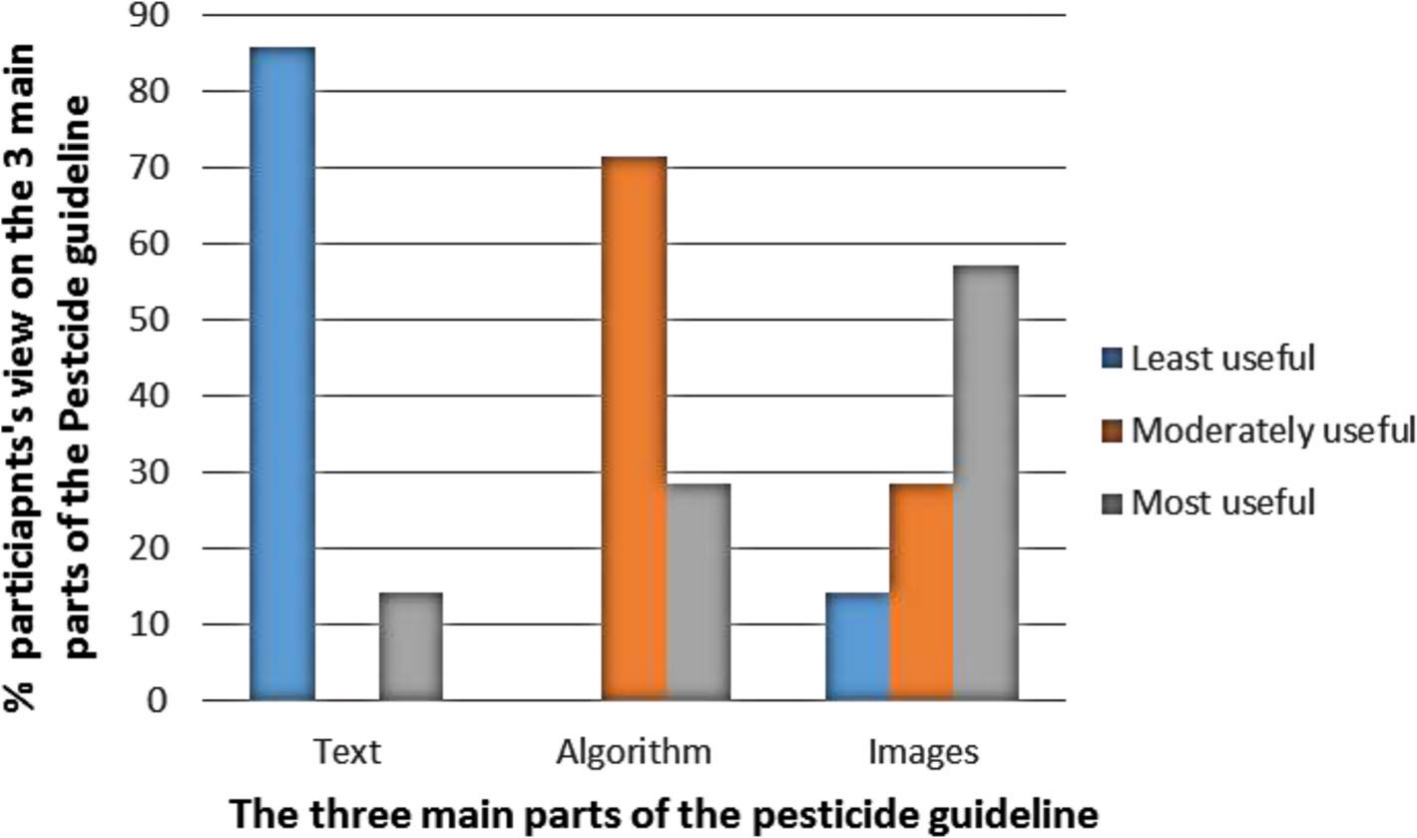
Participants’ perceptions of pesticide guideline components (*N* = 7)

The three main features of the PNG evaluated in this survey included the text format, algorithm for decision-making and pictures. Six of the seven participants that reported using the PNG indicated that the text format displayed on the pesticide guideline was the least useful (Fig. 4). The algorithm for making clinical decisions was found to be moderately useful due to the simplicity of the message, such as outlining the decision-making process when presented with an APP case, especially when the pesticide is unlabelled.

Images displayed on the PNG were shown to be most useful among four of the seven participants. Participants that did not use the images indicated that there were not enough pictures to support them in a clinical diagnosis. Other participants indicated that the images were not useful if patients were unconscious or in unstable condition to assist the doctor in identifying the unlabelled pesticide product responsible for the poisoning.

### Recommendations for improving the PNG-application

Ninety percent of the participants who were unaware of the EM Guidance mobile application, the PNG, or had not used the PNG indicated that they would consider obtaining and using the guideline in the future. When questioned what information they would find useful for the PNG to contain, 74% of the participants provided recommendations, listed in Table 6. Most participants suggested that the guideline should contain clinical-related information, such as antidotes with dosages that may assist HCPs to treat cases of poisoning. This type of information was seen as useful when accessible on a mobile application in a busy ward setting. Other participants valued the idea of having clinical toxidromes (e.g., the cholinergic signs and symptoms that are frequently associated with organophosphate poisoning) to help them to recognize the clinical presentation of APP. Some of the study participants commented on the need for a link to the notification form to automatically notify APPs through the mobile application to streamline this process and make it more likely that HCPs notify more APPs.

**Table 6:** Recommendations for improving the PNG within the EM Guidance mobile application (*N* = 32)

Participants’ recommendations for the PNG within the EM Guidance mobile application	n (%)
Add antidotes with dosages for poisoning situations (e.g. atropine infusion for organophosphate poisoning).	11 (34.4)
Add algorithms for treatment of poison exposures and lists of signs and symptoms/clinical toxidromes related to poisonings.	9 (28.1)
Add link to notify a case to NDOH so as to reduce completing manual paperwork	5 (15.6)
Add mechanism that sends HCPs reminders on how to use PNG and/ or regular updates on the guideline.	2 (6.3)
Provide contact numbers to assist HCPs when enquiring for advice when managing common poisonings (e.g., Poison Information Centre).	2 (6.3)
Add more images.	3 (9.4)

The participants that used the PNG had similar suggestions as in Table 6 (such as a link to notify cases and clinical toxidromes) to those provided by non-guideline users. However, 4 of 7 participants emphasised a need for additional images. One respondent who used the PNG commented:

*“Need a LOT more photos. Clinical treatment and signs and symptoms and investigations need to be included.” [R1]*

## Discussion

The findings of this study illustrate the important role of medical informatics and particularly mHealth application in this case, can play in addressing decision making around diagnosing and treating APP, as well as improving notification. This is a key opportunity particularly as limited training and support provided to medical students and practicing health professionals on APP. While most participants were aware that APP is a notifiable medical condition in SA, that different classes of pesticides that should be notified to the NDOH, most of the participants did not notify APPs. Previous studies in other low- and middle-income countries have identified possible reasons for HCPs not reporting notifiable diseases including busy HCPs having limited time, poor awareness about the prerequisite reporting forms and lack of printed forms [4, 6, 45]. Whereas, possible reasons identified in our study include poor recognition among HCPs of the value of notifying APPs to NDOH and that reporting is a time-consuming obligation. The mobile application, therefore, lends itself to providing an alert system for the need to report. Future mobile applications could include the notification form to simplify the process for reporting.

Our findings showed a significant association between the number of years of work experience of emergency medicine physicians and registrars in relation to knowledge of the legal requirement to report APP. This suggests that experienced emergency medicine physicians and registrars are more likely to report APP as it is expected that healthcare practitioners become more knowledgeable with experience. The question is whether mobile applications that include PNG could reduce the experience period. However, it was unclear why emergency medicine physicians and registrars with more than six years in practice were less knowledgeable on reporting APPs. Perhaps it was a result of notification fatigue, since there are nearly 40 notifiable conditions HCPs need to fill in notification forms for. By including the notification form within a mobile application, perhaps this notification fatigue would be reduced. There was also confusion amongst participants about which pesticide poisonings should be notified, as the current practice was to only report poisonings from organophosphates. Again, the mobile application provides the opportunity to provide accurate information on what needs to be reported, as well as aid in correct diagnosis. Furthermore, future studies are needed to explore why HCPs in general are not reporting APP cases even if they are aware that these must be notified and to evaluate whether mobile applications can address these reasons. This study’s findings suggest an urgent need for educational interventions among HCPs to create awareness and improve notification of APPs, and that there is an opportunity for mobile applications to address this and to provide continual reminders.

The indication is that mobile applications could be useful as continuous training tools in daily medical practice, particularly as most participants own smartphones. This finding is consistent with previous studies conducted in the United States (US) and the UK [32, 46]. The EM Guidance mobile application was one of the most (40%) commonly downloaded medical-related application amongst participants. APP is not only addressed by EM professionals and therefore, information such as the PNG should be added to applications targeting other medical specializations. It is important to note, however, that having a mobile application is not an indication of its use. There was low awareness of the existence of the PNG-application. Therefore, awareness is regarded as the first step that would increase the likelihood of individual use of a specific guideline [47]. Cabana et al. [48] highlight that lack of awareness is one of the factors that hinders the -implementation of guidelines among HCPs. This could explain one of the reasons why, so few participants had used the PNG-application, as its visibility and advertising of information was limited. Since the EM Guidance mobile application was developed in April 2014, there is a possibility that many participants have not yet discovered the guideline. This highlights the need to more proactively advertise the PNG and its relevance. Amongst the participants aware of the guideline, some had never used it as they had only recently downloaded the PNG-application. A similar finding related to the period of dissemination for a guideline was observed in the US, where only 34% of physicians were aware of the existence of the guideline on depression, one year after its publication [49]. Given that APP is a major public health problem, efforts are needed to create a widespread awareness of the PNG-application amongst HCPs through social media, conferences, continuing medical education publications, and programmes or seminars. Coupled with these efforts, APP recognition and reporting should be taught throughout medical undergraduate studies. This would reinforce and ensure that doctors learn how to diagnose and treat APP correctly and the importance of notifying APP to the NDOH. With high mobile ownership among study participants, it showed that PNG-application may support HCPs in reporting more APP cases. Improving the notification process, will not only encourage HCPs to diagnose and notify pesticide poisoning cases more consistently but will also enable NDOH to put measures in place to reduce and prevent APPs.

The Theory of Planned Behaviour (TPB) provided a useful framework to understand participants’ use of PNG-application. According to the TPB, intention to use this guideline or a mobile application in general is influenced by three major constructs: attitude of the HCP, the subjective norm (i.e. perceptions of the views of other HCPs on the adoption of the pesticide notification guideline) and the perceived behavioural control (i.e. HCP’s perception of how difficult or easy it is to use the PNG to diagnose and notify APPs) [37]. Attitude has been considered an important predictor of HCPs’ intentions to follow the recommendations within a guideline [37]. This was observed in our study, where six of the seven participants that used the PNG-application had a good attitude towards the pesticide notification guideline, indicating that the participants intend to continue using it. Similarly, Limbert and Lamb [50] found attitude to be the main prognosticator of intention to utilize the antibiotic guideline. The authors found that doctors had positive attitudes towards the antibiotic clinical guideline because it was useful, and as result they intended to continue using it.

In assessing whether the PNG-application would be used in clinical practice, participants’ self-efficacy was linked to their intention and confidence. Four of the seven participants who had used the PNG previously indicated it improved their confidence in reporting APP cases, particularly those from street or unlabelled pesticides. This finding about self-efficacy is consistent with a study conducted in UK, where Hrisos et al. [51] found that self-efficacy was related to general practitioners’ (GP) intention and confidence to adhere to guidelines in managing upper respiratory tract infection (URTI). Although the number of doctors who used the PNG was small, the indication is that doctors would be more confident to report APP with the support of this mobile application. However, three participants felt that the use of the guideline did not improve their confidence, for reasons that are unknown, as the question was not further queried. It is possible that these participants were familiar with the notification system process, and therefore found no significant change in their confidence in management and notification of APPs through the use of the mobile application. Further research with a larger sample size is needed to investigate these speculations.

The seven participants that used the PNG were comfortable with using the guideline on their mobile device during patient consultations or within the medical setting. In this study, “comfortable” referred to accessing the PNG within the mobile device application in front of patients or in the hospital or other clinical setting. This finding is in contrast with the UK study where participants felt it was unprofessional to use mobile devices during patient consultations or within the hospital setting [32]. The inconsistency in these results is likely due to the cultural concerns related to using a mobile medical application in the hospital, which could differ worldwide. It should be noted that the UK study findings are drawn from one hospital and should not be generalizable to other hospitals or clinical settings.

One reported disadvantage and barrier regarding the use of the PNG-application was that poor network signal reception makes loading of images difficult. This can affect HCPs when they are in the process of showing patients the images to identify which pesticides they were exposed to. The issue concerning mobile applications and slow download speed has also been described in another study [52]. Such an issue may be resolved by ensuring that hospitals have effective Wi-Fi and sufficient coverage to support mobile application use. Another option is to allow for download of the guideline to be used offline and not dependent on the internet each time.

The PNG is the first attempt to support notification and diagnosis of APP. As participants have indicated, there is room for improvement. One of the recommendations given by the PNG-application users was the need for more images or photos of both street and commercial pesticides, which would require more use of bandwidth. This indicates that images are clearer and more useful to HCPs, especially when faced with an APP case from an unlabelled pesticide product. Since mHealth applications are becoming common tools for accessing clinical materials [22], integrating more photos and clinical toxidrome information in the PNG could assist HCPs to promptly diagnose and treat patients suspected of being poisoned. Another suggested recommendation was having a clinical toxidrome in the PNG-application.

HCPs usually have busy schedules within a hectic hospital environment, such as an emergency department. As a result, they may not find time to document in a patient’s record that APP cases need to be notified to the health department. In such clinical practice environments, mobile applications are regarded as convenient tools to accomplish several tasks and provide HCPs with increased efficiencies [22]. Therefore, having a link in the PNG-application that HCPs may use to notify APPs was recommended and, may improve on reporting. Such recommendations are important to consider for increasing the effectiveness of this and future guidelines within mobile application to improve health-care service delivery in general, and to reduce pesticide poisoning specifically.

### Study limitations

There are several limitations of this study. Due to the small sample size and low uptake of the PNG among participants, the generalizability and statistical significance of these study findings are limited. Furthermore, the EM Guidance mobile application was assessed early after its development. It may also be useful to include the PNG in other medical applications besides the EM Guidance mobile application. There was a predominance of participants in this study from the Western Cape in comparison with other provinces. This could possibly be attributed to the fact that the EM profession is well-established in the Western Cape and therefore more of these professionals are found in this region. In addition, recruitment strategies (i.e. involvement of EM physician based in the Western Cape in the recruitment and research process) could have elicited good response and participation rates in the Western Cape in comparison to other provinces, where only emails and phone calls were used. A potential oversight and limitation was that there were no controls built in to reduce the risk of the study participants completing the questionnaire twice or someone else from completing the survey if they were sent the link (e.g., researcher checks of the IP addresses or a password protected questionnaire), although the latter is not seen as a high risk. Finally, individuals who were not familiar with or had not used the pesticide PNG-application were included in the study and therefore were unable to answer some of the questions regarding the guideline. Despite these limitations, this study highlights the need for future research on the use of mobile applications among HCPs in SA, where very few studies have assessed this practice in LMICs.

Future studies using a qualitative methodology (e.g. in-depth interviews) are recommended as this would assist in answering questions of “why” (e.g. why HCPs are not notifying APPs cases) and “how” (e.g. how the PNG-application could be improved). Future studies could also generate more detailed information on the perceptions and experiences of HCPs who have used the PNG-application. It would have been useful to present empirical evidence by comparing aspects of SA context (i.e. health system, cell phone, pesticide use/poisoning, reporting or underreporting) to other related LMICs, but this was beyond the scope of this study.

## Conclusion

The high mobile smart phone ownership among participants indicated that the mHealth platform could provide support, decision making and continued learning to HCPs if mobile applications are well advertised. In addition, integrating mHealth interventions (e.g., EM Guidance mobile application) within the health system could facilitate improvement of HCPs’ reporting of relevant notifiable conditions. Hence governments and industry should support the development of high quality medical mobile applications, in collaboration with HCPs, to address public health challenges and provide information. Lastly, incorporating mobile health applications within healthcare systems and medical schools is a useful means of using technology to support over-burdened medical curriculums and onerous reporting requirements.

## Abbreviations

APP: Acute pesticide poisoning
CEOHR: Centre for Environmental and Occupational Health Research
EM: Essential Medical
GPs: General practitioners
HCPs: Healthcare professional
LMICs: Low- and middle-income countries
mHealth: Mobile Health
NDOH: National Department of Health
OP: Organophosphate
PNG: Pesticide notification guideline
SA: South Africa
SCT: Social Cognitive Theory
SPSS: Statistical Package for Social Sciences
TPB: Theory of Planned Behaviour
UCT: University of Cape Town
UK: United Kingdom
URTI: Upper respiratory tract infection
US: United States
